# Evaluation of being overweight/obese and related sociodemographic factors in 0-5 year age group in Turkey: Turkey Demographic Health Survey 2013 advanced analysis

**DOI:** 10.3906/sag-1808-3

**Published:** 2019-06-18

**Authors:** Asiye UĞRAŞ DİKMEN, Hande KONŞUK ÜNLÜ, Lütfiye Hilal ÖZCEBE

**Affiliations:** 1 Public Health Department, Gazi University Medicine Faculty, Ankara Turkey; 2 Public Health Department, Hacettepe University Medicine Faculty, Ankara Turkey

**Keywords:** Childhood obesity, pediatric obesity, overweight, Turkey

## Abstract

**Background/aim:**

To determine risk factors of overweightness/obesity in children aged 0-5 years in the Turkish population.

**Materials and methods:**

We made advanced analysis using the Turkey Demographic Health Survey (TDHS) 2013 female database, in which data from children aged under five years and their mothers are included. Analyses were performed using weight for height index data. The children were divided into two groups by age as 0–23 months and 24–59 months.

**Results:**

The analysis comprised 2196 children aged under 5 years. Several factors were associated with an increase in overweightness/obesity of children aged under 5 years. Overweight/obesity in children aged 0-23 months was associated with several factors such as age (12–23 months) (OR: 2.89 CI: 1.62-5.13), high birth weight (OR: 2.36 CI: 1.26-4.44), maternal obesity (OR: 2.09 CI: 1.33-3.27), and maternal smoking (OR: 2.07, CI: 1.28-3.33). Overweightness/obesity in children aged 24–59 months was associated with several factors such as education level of the mother (OR: 2.27, CI: 1.08-4.75), consanguineous marriage (OR: 2.86, CI: 1.83-4.47), and which region of Turkey the family lives in (OR: 2.79, CI: 1.53-5.08).

**Conclusion:**

Our results from the TDHS 2013 showed several risk factors of children overweight/obesity. Determining obesity risk factors, monitoring obese children/adults, and providing a multidisciplinary approach to the treatment and prevention of obesity will be useful for the future.

## 1. Introduction

Obesity is a medical condition defined by the World Health Organization (WHO) as abnormal or excessive fat accumulation that presents a risk to health (1). In 2006, the WHO started to use weigth for height index and weight for age index values in the classification of overweightness and obesity according to growth standards for the 0 to 59 months age group. According to these growth standards, overweight is defined as over 2 standard deviations or over the 97th percentile value, and obesity is defined as above 3 standard deviations or the 99th percentile value (2,3).

Childhood overweightness/obesity is one of the most severe public health problems of the 21st century. The prevalence of overweightness/obesity in childhood has increased steadily to alarming levels, and an epidemic approach has begun to be used. This epidemic, described in the childhood age group, concerns the entire world. It is known that 42 million children worldwide under the age of 5 years in 2010 were overweight and obese (4). It is estimated that in 2025, a total of 70 million children aged under 5 years will be affected if the trend of increasing overweightness/obesity continues in children (5). Overweight/obese children are also more likely to become overweight/obese during adulthood. In children with this risk, noncommunicable diseases, particularly diabetes mellitus and cardiovascular diseases, increased psychosocial health problems, increased risk of middle-aged deaths, and lower success rates in education and workplaces were observed (6).

Overweightness and obesity in children are also important problems for the Turkish population. Despite this, there has been limited research to reveal the factors associated with the incidence of overweightness/obesity in Turkey. The Turkey Demographic Health Survey (TDHS) is one of the critical studies that showed overweightness/underweightness in children aged under five years in 2013, and the percentage over the 2 standard deviations according to height in the 0–5 years age group was 10.9% (3). In a study conducted in Turkey in 2014, 8.5% of children aged between 0–5 years were reported as obese, and 17.9% were considered overweight (7).

Many factors cause overweightness and obesity problems in children. In different studies, it was found that factors such as sedentary lifestyle, high birth weight, obesity history in the family (overweight mother), smoking around the children, low or high-income level, low or high education level, and inadequate breastfeeding were effective in overweightness/obesity (8). In the childhood age group, especially in children aged younger than 12 years, drug treatment is not recommended for overweightness and obesity treatment, and surgical procedures are considered as a last resort in unresolved cases. Therefore, it is necessary to take preventive measures by determining the related risk factors in the first four years of life, which is the basis for fighting childhood obesity (9,10).

Childhood obesity is a serious problem because of its high frequency, its impact on many aspects of the adult population. However, it’s a preventable health problem with short and long-term interventions. When taking into consideration the rising rate of the childhood obesity in the world, it is thought that countrywide research is regional and does not adequately reflect risk factors in a country as a whole. In TDHS 2013, overweightness/obesity data were presented for the first time in children. The identification of the risk factors will guide both the prevention of obesity and similar studies because there are differences in risk factors among societies. The purpose of this study was to assess overweightness/obesity in the 0-5 years (0-59 months) age group and related factors based on the TDHS 2013 data.

## 2. Materials and methods

This study is a secondary data analysis of the 2013 database of the TDHS, which is conducted every five years by Hacettepe University Institute of Population Studies (3). The TDHS 2013 database, which is open to general use, was obtained from the Hacettepe University Institute of Population Studies. Data from children aged under five years were included in the TDHS 2013. The database of women was also evaluated within the scope of the study. Mothers of children aged 0 to 59 months were selected from the TDHS women’s database. As the analysis was done through the women’s database, younger children aged 0–59 months of women with more than one child were included in the study and the final analysis was performed with 2196 children.

Analyses were performed using weight for height index data. According to the recommendation of the WHO (3), children with +2 standard deviations were considered overweight/obese. The overweight/obese data in the database are presented as Z-scores; values over +200 correspond to +2 standard deviations. Obesity of the mother was considered as BMI ≥ 30 kg/m2 (3). In the analysis of the risk factors from the TDHS 2013 female database, data related with only children under the age of five years and women’s data related to overweightness/obesity in children (e.g., household income level, parental education status, type of residence, mother tongue) were used. Figure shows the flow diagram of the study. 

**Figure:**
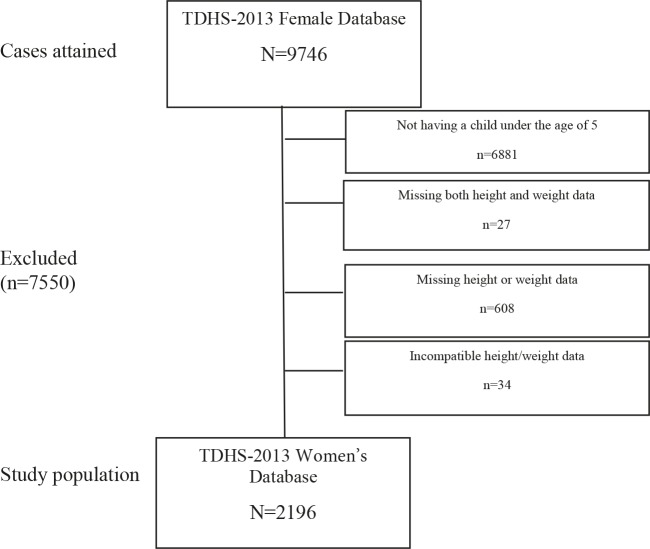
Flow diagram of the study.

### 2.1. Statistical analysis

Statistical analysis was performed using the “Complex Samples” module in IBM SPSS version 23.0 (IBM Corp, Armonk, NY, USA) because a weighted, multi-stage, stratified cluster sampling approach was used in the TDHS-2013 study. The children were divided into two groups by age as 0–23 months and 24–59 months. All analyses were performed for each age group. For the study sample characteristics, categorical variables are reported as frequencies and weighted percentages. Chi-square tests were conducted to examine differences between obesity status of child and other categorical variables. If the result of the Chi-square test was found as statistically significant, standardized residual values were examined to determine which variables caused the differences. In each child age group, binary logistic regression was constructed to identify the relationship between child overweight/obesity status and the following explanatory variables: sex of child, age group of child, size of child at birth, still breastfeeding, obesity status of mother, education level of mother, education level of father, smoking status of mother, parents related, level of income, region, and type of residence.” In the backward model, variables were included as independent variables if they were significant between 0.05–0.20 level or were found as significant according to Chi-square test. While performing multiple logistic regression, the listwise deletion method was used to handle missing observations. P value below 0.05 was accepted as significant.

## 3. Results

Sociodemographic features (age, sex) and nutrition-related features (birth weight, birth order, and breastfeeding-related characteristics) of overweight/obese children are presented in Table 1. The frequency of obesity in children aged 12–23 months and then 6–11 months were significantly higher than in other ages (P < 0.001). The frequency of obesity was found to be significantly higher in children who were above average birth weight (P = 0.003) among those aged 0 to 23 months. The same relationship was found statistically significant in the 24–59 months age group (P = 0.033). There was no relationship among birth order, the status of being breastfed after delivery, giving sugary water, giving formula, giving milk other than breast milk, and bottle-feeding and obesity. The frequency of obesity was found to be significantly lower in children who were still being breastfed among those aged 0 to 23 months.

**Table 1:** Overweight/obesity status of children aged 0–59 months by sociodemographic and nutrition-related characteristics, Turkey, 2012.

	0–23 months old overweight/obese children			24–59 months oldoverweight/obese children		
	%1	n2	P	%1	n2	P
						
Sex						
Boy	14.6	536	0.446	11,4	636	0.204
Girl	12.8	511		8,8	513	
Months						
0-5 months	6.4*	213	<0.001			
6-11 months	11.9	301				
12-23 months	17.7*	533				
24-36 months				12.3	451	0.224
37-48 months				9.2	378	
49-59 months				8.7	320	
Birth weight						
Low	9.5	250	0.003	5.1*	266	0.033
Normal	13.5	635		11.9	692	
High	22.1*	156		10.9	190	
Birth order						
1st child	15.0	298	0.627	13.0	285	0.112
2nd-3rd child	13.4	554		10.1	647	
4th-5th child	14.7	127		6.7	135	
6th+ child	9.7	68		4.1	82	
Breastfeeding after delivery						
Immediately	15.0	597	0.220	10.0	639	0.588
In an hour	10.9	57		12.4	63	
After an hour	11.1	255		8.7	325	
After a week	16.8	119		15.0	99	
Never	---	18		12.5	2	
Still breastfeeding						
No	18.0*	338	0.030	10.2	1056	0.923
Yes	12.2	686		10.7	68	
Taking milk except for breast milk in the first three days AD						
No	14.3	1012	0.143	10.3	1119	0.402
Yes	---	15		---	6	
Giving formula in the first three days AD						
No	14.0	757	0.962	9.8	907	0.479
Yes	14.2	270		11.6	218	
Giving sugar water in the three days AD						
No	14.2	976	0.537	10.2	1066	0.923
Yes	11.2	51		9.6	59	
Bottle-feeding before the interview night						
No	13.5	474	0.845	9.1	780	0.106
Yes	14.0	573		12.6	368	

1Row percentage of overweight/obesity status. 2Total unweighted counts. AD: After delivery. *Statistically significant cells

The descriptive characteristics of parents of obese children are shown in Table 2. In the 0–23 months age group, there was no relationship between maternal age, parental consanguinity, mother tongue, and obesity. In children aged 24 to 59 months, obesity was found to be higher only in consanguineous marriages; there was no difference in terms of other parental features. In contrast, in children aged 0–23 months, obesity was associated with many parental characteristics. Higher educational level of the mother, maternal smoking, and the mother’s obesity were associated with obesity of children. In addition, a lower education level of the father and higher welfare level of parents were associated with obesity in the children.

**Table 2:** The descriptive characteristics of parents of overweight/obese children, Turkey, 2012.

	0–23 months old overweight/obese children			24–59 months old overweight/obese children		
	%1	n2	P	%1	n2	P
Maternal age (years)						
15-19	9.8	51	0.706	22.2	5	0.542
20-29	13.7	584		11.3	446	
30-39	14.5	384		10.0	592	
40-49	11.5	28		6.7	106	
Status of mother education						
No education	8.5	161		4.2	150	0.133
Elementary school	10.9	366		11.8	532	
Secondary school	16.8	409		10.3	351	
High school and above	17.4*	111	0.015	10.0	116	
Status of father education						
No education	19.9	45	0.002	6.0	43	0.501
Elementary school	8.4*	394		8.8	440	
Secondary school	16.7	441		11.1	495	
High school and above	16.7	165		12.6	167	
Welfare level						
Poor	10.3	565		8.3	569	0.117
Normal	13.2	204		9.2	239	
Rich	18.6*	278	0.012	13.0	341	
Consanguineous marriage						
Yes	13.9	313	0.938	15.7*	297	0.002
No	13.7	733		8.5	852	
Mother tongue of parents						
Turkish	14.3	676	0.179	11.2	842	0.258
Kurdish	12.4	314		8.4	266	
Arabic	5.4	46		---	26	
Others	36.9	11		---	15	
Maternal smoking status						
Smoking	24.3*	178	<0.001	11.0	260	0.657
Not smoking	11.3	869		9.9	888	
Status of mother obesity						
Obese	21.6*	148	0.003	9.8	154	0.840
Not obese	12.3	888		10.4	988	

1Row percentage. 2 Total unweighted count. *Statistically significant cells.

Table 3 presents area and region distrubition of obesity. Although there was found no difference between regions in children aged 0 to 23 months, the frequency of obesity was found higher in children from urban areas. In children aged 24 to 59 months, the least amount of obesity was in the Eastern Anatolian region and highest in the Middle Anatolian region. There was no difference in the frequency of obesity according to settlement.

**Table 3:** Regional and area distribution of overweightness/obesity, Turkey, 2012.

	0–23 months old overweight/obese children			24–59 months old overweight/obese children		
	%1	n2	P	%1	n2	P
Regions						
West	16.5	187	0.105	12.3	254	0.001
South	16.1	167		6.4	160	
Middle	15.3	171		13.7	217	
North	11.2	130		10.4	179	
East	8.2	392		5.2*	339	
Residence						
Urban	15.2	755	0.007	10.3	829	0.880
Rural	8.2*	292		10.0	320	

1Row percentage. 2 Total unweighted count. *Statistically significant cells.

As shown in Table 4, the effects of different variables on obesity frequency were determined. In the model, which was constructed for children in the 0–23 months age group and in children aged over 1 year, the frequency of obesity was found as 2.8 times high compared with babies in their first 6 months. Obesity was 2.3 times more frequent in children with high birth weight than in children with low birth weight. The frequency of obesity was twice as high in children with obese mothers and twice as high in children with smoking mothers. In the model, which was constructed for children over 2 years old, the frequency of obesity was 2.8 times higher in children whose parents had a consanguineous marriage than in children whose parents were not in a consanguineous marriage. In addition, the frequency of obesity was 2.7 times higher in children living in the western and middle regions than the children living in the eastern region.

**Table 4:** Various independent factors associated with childhood overweight/obesity, Turkey, 2012.

	0–23 months old children1,2			24–59 months old children3,4		
	OR	CI	P	OR	CI	P
Age group (months)						
0-5 months	Ref.		<0.001			
6-11 months	1.933	0.947-3.948				
12-23 months	2.891	1.628-5.133				
Birth weight						
Low	Ref.		0.022			
Normal	1.382	0.853-2,239				
High	2.368	1.263-4.440				
Status of mother obesity						
Obese	2.092	1.338-3.272	0.001			
Not obese	Ref.					
Status of mother education						
No education				Ref.		0.018
Elementary school				2.276	1.089-4.757	
Secondery school				1.387	0.593-3.241	
High school and higher				0.884	0.323-2.423	
Maternal smoking status						
Smoking	2.071	1.287-3.334	0.003			
Not smoking	Ref.					
Consanguineous marriage						
Yes				2.865	1.835-4.472	<0.001
No				Ref.		
Regions						
West				2.776	1.486-5.186	0.001
South				1.346	0.683-2.654	
Middle				2.796	1.538-5.085	
North				1.823	0.926-3.591	
East				Ref.		

1Percentage of correct classification: 85.9%. 2Adjusted for sex of child, still breastfeeding, status of mother education, status of father education,consanguineous marriage, welfare level, regions and residence. 3Percentage of correct classification: 89.7%. 4Adjusted for sex of child, age group (months), birth weight, status of mother obesity, status of father education, maternal smoking status, welfare level and residence.

## 4. Discussion

Overweightness and obesity are considered to be a worldwide epidemic, the prevalence of which has dramatically increased among children during the last decades (11). The prevalence of overweightness and obesity varies across countries and years of study. The national prevalence obesity of United States of America was found as 7.2% in 1988, 13.9% in 2004, and 9.4% in 2014 (12). The prevalence of obesity in preschool children in China was reported as 3.9% in 1992 and 5.4% in 2002 (13). In Brazil, the prevalence of obesity increased from 6.7% to 9.3% in children aged under five years (14). In a study in Kuwait, the prevalence of preschool obesity was found as 8.2% (15). Similar findings were also obtained in a comprehensive study that used data from 450 cross-sectional surveys from 144 countries (16). In that study, the prevalence of childhood overweightness and obesity in pre-chool children was reported as 4.2% in 1990 and 6.7% in 2010. These studies suggest that childhood obesity tends to increase. The prevalence of overweightness and obesity in children aged under five years was found as 10.9%–17.9% in Turkey (6, 7). According to TDHS 2013 findings, one out of every ten children was overweight/obese (6). In the Childhood Obesity Surveillance Initiative-Turkey (COSI-TR) study, the prevalence of obesity was found as 22.5% in children aged 7–8 years (17). It is thought that if obesity/overweightness is not prevented, it will increase later in Turkey. Identified risk factors should be considered for the prevention of overweightness/obesity. The aim of this study was to determine factors associated with overweightness/obesity. 

Many studies have reported a positive association between high birth weight and obesity in older children and adults (18–21). Also, similar results were found in children aged up to 7 years (22, 23). A systematic review conducted by Martins et al. (over 20 studies) in 2016 showed that there was a positive association between birth weight and childhood obesity (22). Our research also supports this finding. In the present study, the frequency of obesity was 2.3 times more frequent in children with high birth weight than in children with low birth weight. These results were interpreted that obesity became a chronic process if obese children with high birth size were not treated. Besides, studies have reported that low birth weight was protective against the development of obesity (23).

The WHO recommends exclusive breastfeeding in the first six months, then breastfeeding to 24 months with a supplementary diet. In children aged 0–23 months, a higher prevalence of obesity was found in those who were not breastfed. Breastfeeding is a protective factor for obesity of early childhood (24). Armstrong et al. reported that breastfeeding reduced the risk of childhood obesity in a study conducted with 32,200 children aged 39–42 months (24). It is thought that adherence to the recommendations of the WHO regarding breastfeeding would reduce the prevalence of obesity.

Many factors related to mothers have been associated with obesity in children. Lamerz et al. reported a strong relationship between mother’s high educational status and obesity in children. However, Fitzgibbon et al. (2005 and 2006) found no statistically significant relationship between the mother’s education and the prevalence of obesity in children aged under 60 months (25, 26). On the contrary, Felisbino-Mendes et al. reported that there was a positive relationship between the level of maternal education and obesity in children aged under 60 months. The frequency of obesity increased as the mother’s education level increased (27). In our study, there was a difference in childhood obesity related with the mother’s education levels; it was observed that the frequency of obesity increased as the mother’s education level increased. This situation may be associated with an increase in the socioeconomic level of the family due to the increase in the education level of the mother. As the socioeconomic level increases, unhealthy diets may also increase. In addition, this may be related to the fact that highly-educated mothers perceive their children as overweight. Baugchum et al. emphasized that preventing obesity/overweightness in preschool children could not succeed without understanding their mother’s perception of the problem when treating the obesity problem (28). Prenatal exposure to tobacco can lead to life-long effects as a result of DNA methylation (29). Von Kries et al. reported that smoking during pregnancy caused childhood obesity by affecting babies in utero (30). Also, mothers who smoke may also be less likely to monitor their children’s health. The results of our study support a rising prevalence of obesity in children whose mothers smoke (31). Another factor related to maternal characteristics about childhood obesity is maternal obesity status. Maternal obesity increases the risk that children will become obese/overweight (32). The findings of our study support this finding. A number of mechanisms could be responsible for the links between childhood obesity and maternal obesity. Sloboda and Vickers reported that obesity of the mother might transfer to the child via nonMendelian mechanisms (33). Lesseur et al. reported that the obese mother might be effective by disrupting the leptin DNA methylation in the child (34). The family characteristics (lifestyle, traditional behavior, and health behaviors) in which children live can influence children’s behaviors and health outcomes. There is a need for more research in this area.

There are a limited number of studies in the literature about the level of children’s father’s education and its relationship with childhood obesity. A recent study by Savaşhan et al. in 2015 reported that there was no relationship between father’s education and obesity in school aged children (35). In contrast, Sarrafzadegan et al. observed that higher levels of education of the father were associated with obesity (36). In our study, there was no linear correlation between the father’s education level and frequency of obesity. 

A systemic study showed no clear relationship between socioeconomic level and childhood overweightness/obesity (37). On the contrary, Vitolo et al. reported that a high socioeconomic level was associated with overweightness in children aged under five years (38). The results of our study are consistent with those of Vitolo et al. This may be caused by children with higher socioeconomic levels consuming high-calorie food and avoiding physically challenging tasks.

Joens-Matre et al. suggested that there were rural-urban differences in obesity of children and adolescent (39). In the literature, there are no consistent findings that residence factors are a risk for obesity in children aged 0-5 years. In this age group, different studies have reported that obesity is more common in both urban and rural areas (7, 40). De Arruda Moreira et al. reported that children aged under five years did not differ in urban and rural regions concerning overweightness and obesity (23). In the present study, urban residence was found higher in children aged 0-2 years, whereas it was found similar in children aged 2-5 years. Despite the fact that high education was more common in the urban residence group, the rural-urban difference in the development of overweightness/obesity cannot be solely explained by the differences in the educational level of parents. The educational level of the father and mother were not parallel in our study. This result may be due to urbanization; the difficulty of accessing places for physical activity and easier access to high-calorie foods. Also, we found that obesity increased as the level of income increased in children aged 0 to 2 years. The fact that the welfare of the people living in the city is better could explain this situation.

In conclusion, further analysis found that child characteristics, parents’ characteristics and type of residence were effective on childhood obesity in Turkey. It would be beneficial to identify obesity risk factors, monitor obese patients, and present a multidisciplinary approach to the treatment and prevention of obesity. Both family and health professionals may make important contributions to treatment and prevention of obesity. Obtaining accurate information about obesity by parents may be possible through health education. There is a need for further studies to identify the environmental and cultural factors associated with overweightness/obesity.
